# Management of intrathoracic phosphaturic mesenchymal tumor by nonintubated uniportal video‐assisted thoracic surgery in a fragile patient

**DOI:** 10.1002/cnr2.1500

**Published:** 2021-08-05

**Authors:** Michele Ferrari, Alessandro Palleschi, Francesca Bartoli, Federico Polli, Elisabetta Armiraglio, Antonina Parafioriti, Giorgio A. Croci, Davide Tosi

**Affiliations:** ^1^ Department of Pathophysiology and Transplantation University of Milan Milan Italy; ^2^ Thoracic Surgery and Lung Transplantation Unit Fondazione IRCCS Ca' Granda—Ospedale Maggiore Policlinico Milan Italy; ^3^ Department of Rheumatology Gaetano Pini Institute Milan Italy; ^4^ Department of Anaesthesia Critical Care and Emergency Medicine, Fondazione IRCCS Ca' Granda—Ospedale Maggiore Policlinico Milan Italy; ^5^ Department of Pathology ASST‐PINI‐CTO Centro Specialistico Ortopedico Traumatologico Gaetano Pini Milan Italy; ^6^ Division of Pathology Fondazione IRCCS Ca' Granda, Ospedale Maggiore Policlinico Milan Italy

**Keywords:** hypophosphatemia, NITS, phosphaturic mesenchymal tumors, uniportal VATS

## Abstract

**Background:**

Phosphaturic mesenchymal tumors are rare neoplasms, frequently presenting with osteomalacia. These neoplasms usually grow at a slow rate and are associated with unspecific symptoms.

**Case:**

In this study, we present the case of a 70‐year‐old woman who had been suffering from musculoskeletal pain, hypophosphatemia, and spontaneous fractures. Positron emission tomography with Gallium showed increase uptake in a subpleural lesion.

**Conclusion:**

The patient underwent surgical excision of the subpleural lesion with a non‐intubated uniportal video‐assisted thoracoscopic surgery approach.

## 1 INTRODUCTION

Phosphaturic mesenchymal tumors (PMTs) are rare mesenchymal tumors that produce a phosphate substance, fibroblast growth factor‐23 (FGF23), causing systemic phosphate depletion and leading to oncogenic osteomalacia. Osteomalacia is linked to an increased circulating level of FGF‐23, produced by the PMT, which reduces renal reabsorption of phosphate and inhibits 25‐hydroxyvitamin D1‐α‐hydroxylase function.^1^, ^2^ Significant factors contributing to late diagnosis are the slow growth and small size of the mass, as well as non‐specific clinical characteristics (bone pain, spontaneous fractures, muscle weakness, and impaired gait). Patients are extremely fragile because of the substantial number of bone fractures they sustain.^3^ Usually, surgical excision of the tumor is the treatment of choice for PMT, but surgery is sometimes contraindicated due to several coexisting comorbidities.^4^

Historically, thoracic surgery has always been performed under general anesthesia using a double‐lumen endotracheal tube, which allows one‐lung ventilation. In recent years, new anaesthesiologic techniques have been developed with a specific interest in nonintubated (spontaneously ventilated) procedures. Nonintubated thoracic surgery (NITS) is performed under loco‐regional anesthesia, specifically intercostal/interpleural/paravertebral blocks or epidural anesthesia. The development of minimally invasive techniques like multiportal and uniportal video‐assisted thoracic surgery (VATS) has encouraged improvements in postoperative pain management, reduction of postoperative complications and hospital stay. The combination of the uniportal approach and nonintubated anesthesia results in a minimal impact on the patient.^5^, ^6^ We present the clinical case of a symptomatic intrathoracic PMT, treated with non‐intubated uniportal VATS.

## 2 CASE REPORT

**FIGURE 1:**
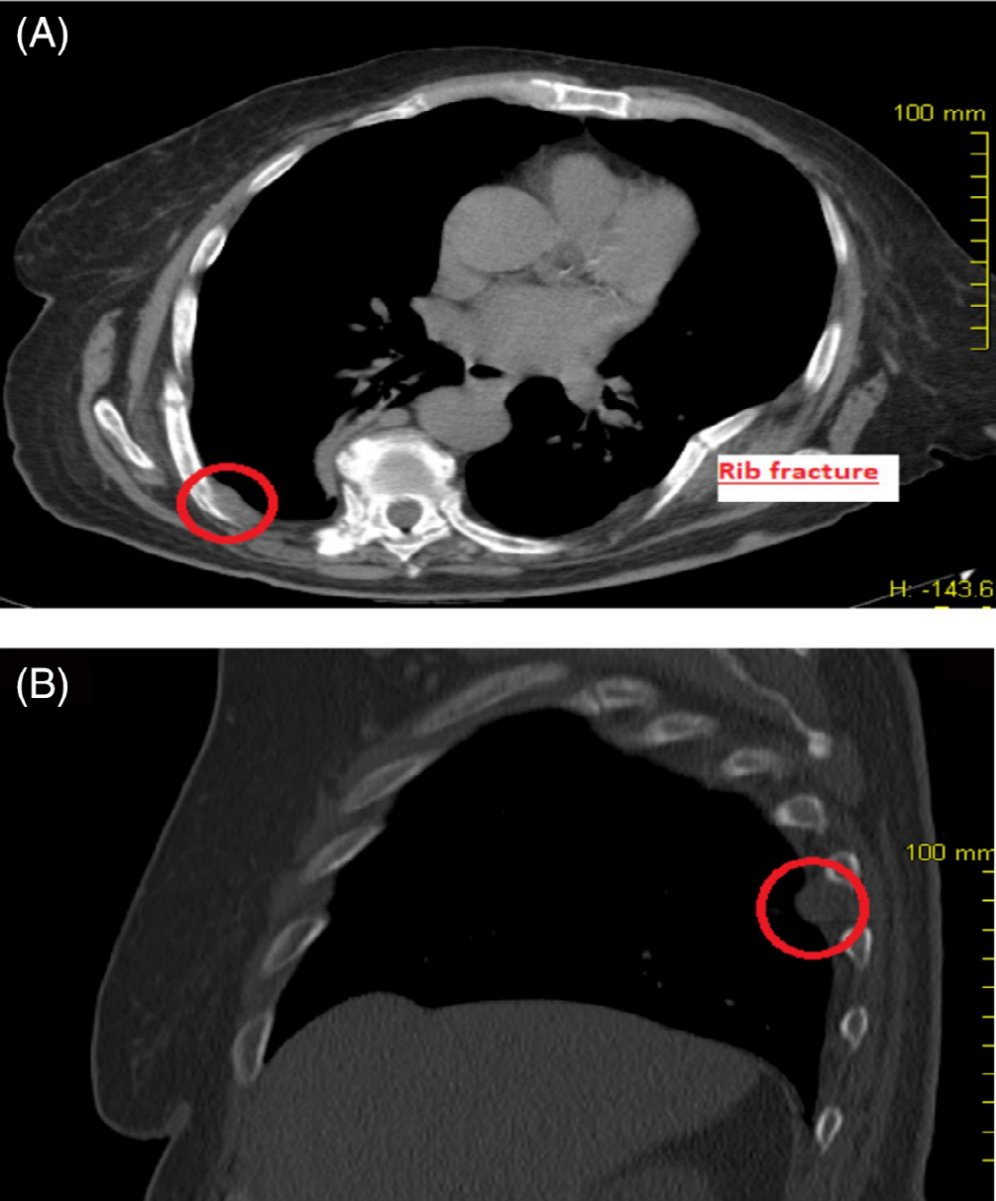
(A, B) CT images of intrathoracic phosphaturic mesenchymal tumor and rib fracture

**FIGURE 2:**
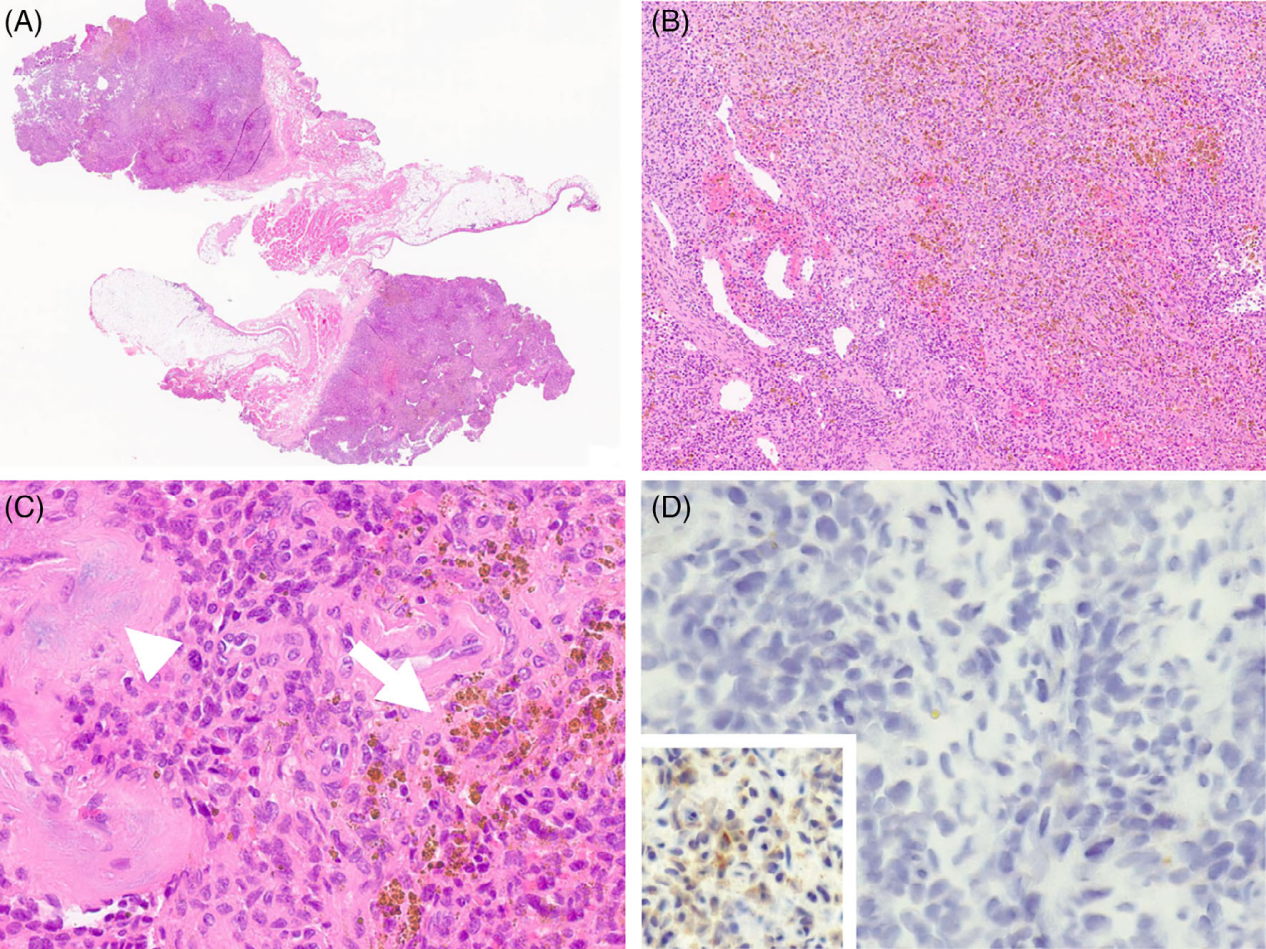
(A–D) Histologic Panel of phosphaturic mesenchymal tumor. At scanning magnification (2A. HE), the lesion appears as a mass forming proliferation with a grossly pushing border toward the adipose tissue of the chest wall. At higher magnification (2B. HE, 10×) the lesion consists of bland looking ovoid to spindle cells, with an unremarkable mitotic rate and absence of necrosis, growing in a diffuse fashion with irregularly intermixed hematic vascular structures. The detail (2C. HE, 40×) underlines the presence of the accumulation of hemosiderin laden macrophages (arrow) and of small foci of myxoid degeneration with finely granular calcifications (arrowhead). Immunohistochemical stain with anti‐FGF23 antibody (2D. 40×) resulted negative, as compared to the positive control (inset)

A 70‐year‐old female patient had been suffering from bone pain, asthenia, and multiple fragility fractures of the lower limbs, pelvis, ribs, and spine for several years. The patient was in poor clinical conditions due to prolonged bed rest because of repeated bone fractures. Although numerous neurological and rheumatological evaluations had been previously performed, all clinical and serum tests gave nonspecific findings, and a definitive diagnosis was not obtained. During her last hospitalization at the Rheumatology Department of the Gaetano Pini Hospital (Milan, Italy), the following laboratory abnormalities were found: low serum phosphorus (Table 1), an improperly normal calcitriol (1,25[OH]2D) and an increased urinary phosphate excretion detected by a reduced tubular maximum phosphate reabsorption per glomerular filtration rate (TMP/GFR).^7^, ^8^ Once these biochemical changes were highlighted, TIO was suspected. A subsequent 68‐Gallium DOTATATE positron emission tomography (^68^Ga‐DOTATATE PET/CT) confirmed a pathological uptake in a subpleural lesion near the eighth right rib (Figure 1(A), (B)). The patient was then transferred to our department for proper management. The clinical case was presented at the multidisciplinary tumor board meeting and a surgical resection indicated. Due to bone fragility, the passive mobilization of the patient was deemed too risky; to allow active positioning on the operative table, a nonintubated surgical resection of the tumor was planned. In the operative room, after patient's self‐positioning in the lateral decubitus position, general anesthesia was induced with propofol (using a target‐controlled infusion) and fentanyl; the airway was secured with a laryngeal mask (Ambu® AuraGain™), and spontaneous breathing of 100% oxygen was maintained throughout the procedure. We performed a uniportal VATS resection with the surgeon and the assistant facing the patient. After local anesthesia of the skin (5 ml of lidocaine 2%), a 4‐cm utility muscle‐sparing incision was performed on the 5th intercostal space and a wound protector (Alexis®, Applied Medical, USA) was positioned; the thoracoscope was always positioned at the upper end of the incision port. After gaining access to the thoracic cavity, four intercostal nerves were blocked with local anesthetic (each with 3 ml of ropivacaine 7.5 mg/ml) under direct thoracoscopic vision and the vagus nerve was also blocked near Barety's space (5 ml of ropivacaine 7.5 mg/ml) to reduce the cough reflex. The radical resection of the lesion was carried out without intraoperative complications (Video S1). The postoperative course was uneventful. Rapid mobilization soon followed. The size of the surgical sample was 30 × 15 × 10 mm. Histologic examination revealed a patternless proliferation of ovoid to spindle cells, featuring minimal atypia and unremarkable mitotic rate, in the absence of necrosis. Immunohistochemical analysis, as routinely performed via automated staining on a Dako OMNIS (Agilent, Carpinteria, CA) platform, disclosed a “vimentin‐only” phenotype, without expression of substantial negativity for markers of vascular/endothelial (ERG, CD31, CD34, and podoplanin), muscular (smooth muscle actin, desmin) and neural (S100) differentiation, and low and high molecular weight cytokeratins (Figure 2A–C). In addition, immunohistochemical analysis of FGF23 was performed using a commercially available anti‐FGF23 antibody (1:80 dilution; antibodies‐online, Aachen, DE), which turned negative (Figure 2D). In the absence of sufficient criteria for the alternative diagnosis of hemangiopericytoma or solitary fibrous tumor, the benign histologic picture together with the clinical history and the laboratory findings prompted a diagnosis of PMT.^9^ The normalization of phosphate levels occurred on 14th postoperative day. During the 1‐year follow‐up, the patient showed normalization of the electrolyte concentrations, exhibiting no symptoms nor any sign of endothoracic lesions or recurrences noted on repeated CT scans. Due to the remission of the clinical disease, radiological follow‐up was stopped and only 6‐monthly biochemical monitoring was provided.

**TABLE 1:** Preoperative values of electrolytes and hormones

Serum phosphorus	0.8 mg/dl
24 h urinary Pi	191 mg
TMP/GFR	0.2 mg/dl
Serum calcium	7.7 mg/dl
24 h urinary Ca	32 mg/24 h
Serum ALP	215 U/L
Intact PTH	153 pg/ml
1,25(OH)2D	45 ng/L
Albumin	3,4 g/dl
Albumin‐corrected serum calcium	8,2 mg/dl

*Note: Reference values*. Serum phosphorus: 2.5–4.6 mg/dl; 24 h urinary Pi: 400–1000 mg; TMP/GFR: 2.3–4.3 mg/dl; Serum calcium: 8.4–10.2 mg/dl; 24 h urinary Ca: 100–300 mg/24 h; Serum ALP: 30–130 U/L; Intact PTH: 15–65 pg/ml; 1,25 (OH)2D: 25–86 ng/L.

## 3 DISCUSSION

PMT is tumor of the soft tissue and bone that, in some cases, appears as a paraneoplastic pathologic TIO. This condition was first described by McCance in 1947.^10^ PMTs are classified into four subtypes: phosphoric mesenchymal connective tissue variant (PMTMCT), osteoblastoma‐like variant, ossified fibroids‐like variant and nonossifying fibroma‐like variant. The PMTMCT variant is the most common, making up 70–80% of reported cases.

According to the literature, 46% of the cases of TIO occurred in females and 54% in males, with an average age of 45.3 years at the time of diagnosis.^11^

In the majority of patients (57%), the tumor arises in bones, while in the remaining 34% cases it originates in soft tissues.^12^

Tumors are usually located in the femur (22.7%), skull (20.7%), ankle and foot (8.8%), pelvis (8.2%), tibia or fibula (6.5%), and arm (6.5%). The intrathoracic localization is uncommon.^4^

In order to locate the culprit tumour, the whole body functional imaging tests should be conducted first. 18‐fluoro‐deoxy‐glucose (F‐18 FDG) Positron Emission Tomography/Computed Tomography (F‐18 FDG PET/CT) is a very sensitive but not very accurate diagnostic tool.^13^

The best accurate imaging currently available involve the use of radiolabelled tracers for somatostatin receptors (SSTRs) commonly expressed by PMTs^14^; 68Ga‐DOTATATE PET/CT, for the highest sensitivity and specificity, is considered the gold standard for tumor localization.^15^ Magnetic resonance imaging can effectively confirm the tumor site and the margins with surrounding tissues.^16^ Surgical excision with adequate margins is the treatment of choice for these patients because distant metastases are very rare.^4^, ^10^ Within a few days after surgical resection, the serum markers usually return to baseline, while few months are necessary for the complete resolution of the symptoms.^2^ Radiotherapy is a valid alternative when resection is incomplete or for patients who are considered not fit for surgery.^17^ Caudell et al. observed that combined therapy (surgery and adjuvant radiotherapy) had better control on disease than radiotherapy alone.^18^ Patients with PMT are very fragile and extremely compromised because of long periods of bed resting for recurrent pathological bone fractures and pain. The surgical risk is high and the operation may be contraindicated and comorbidities can play a relevant role in case of intrathoracic growth, however treatment should be mandated as removal of the lesion commonly leads to normalization of laboratory anomalies and, along the follow up, to normalization of the clinical condition. In the case of PMT, manifestations are postulated to be dependent on the production of FGF23. Lack of FGF23 detection via immunohistochemistry did not rule out a diagnosis of PMT in our cases: such a feature is well described^3^, ^17^ and it may depend on the levels of FGF23 secretion by the tumour cells, which may fall below the sensitivity of the commercially available antibodies. For these reasons, NITS was our first choice. Historically, the development of thoracic surgery followed the introduction of mechanical ventilation in the 1960s. Since the early 2000s, interest in NITS procedures has grown in the surgical community; this approach was initially applied to simple procedures only (pleural biopsy or resection of pulmonary bullae). In 2004, Pompeo et al. reported a case series of awake thoracoscopic resections of solitary pulmonary nodules under epidural anesthesia, demonstrating the feasibility and safety of this technique.^19^ In 2007, Al‐Abdullatief et al. reported the first nonintubated VATS lobectomy in an awake patient.^20^

Since then, many authors have reported the combination of NITS with thoracoscopic techniques. Rocco et al. have described the technique of resection of pulmonary nodules in awake patients, even in an ambulatory setting.^21^ The combination of NITS and the evolution of VATS, specifically of the uniportal approach, have minimized the invasiveness of the procedures and reduced the hospital stay.^22^ Common side effects of tracheal intubation and general anesthesia (intubation‐related airway trauma, ventilator‐induced lung injury, and residual neuromuscular blockage) are less frequent as well, allowing early recovery after the operation. It is relevant to point out that avoiding general anesthesia and one‐lung mechanical ventilation could result in a better physiological inflammatory response, leading to oncological advantages in neoplastic patients.^23^

Despite all these advantages, NITS has some contraindications such as obesity, neurological conditions, uncontrolled gastroesophageal reflux, central hypoventilation syndrome, persistent cough or mucus retention, hemodynamic instability, and severe hypoxia/hypercapnia.^5^Therefore, a careful selection of patients is mandatory to obtain satisfactory results.

This case report describes a classic case of osteomalacia induced PMT. By using the NITS technique, it was possible to have the patient autonomously positioned on the operating table, reducing the risk of further bone fractures during lateral decubitus mobilization. A minimally invasive uniportal approach was used to reduce peri‐ and postoperative pain and the risk of iatrogenic rib fractures.

The key point of this case report is to highlight the effectiveness of combining the NITS technique with the VATS uniportal approach, which has proven to be an optimal strategy, minimizing peri‐operative complications and hospital time.

## CONFLICT OF INTEREST

The authors have stated explicitly that there are no conflicts of interest in connection with this article.

## AUTHOR CONTRIBUTIONS

**Michele Ferrari:** Conceptualization; formal analysis; methodology; resources; writing‐original draft; writing‐review & editing. **Alessandro Palleschi:** Conceptualization; formal analysis; methodology; resources; supervision; writing‐original draft; writing‐review & editing. **Francesca Bartoli:** Conceptualization; formal analysis; methodology; resources; supervision; writing‐original draft. **Federico Polli:** Conceptualization; data curation; methodology; writing‐original draft. **Elisabetta Armiraglio:** Conceptualization; formal analysis; investigation; resources; writing‐original draft; writing‐review & editing. **Antonina Parafioriti:** Conceptualization; formal analysis; investigation; methodology; resources; writing‐original draft; writing‐review & editing. **Giorgio Alberto Croci:** Conceptualization; formal analysis; methodology; resources; writing‐original draft; writing‐review & editing. **Davide Tosi:** Conceptualization; formal analysis; methodology; project administration; supervision; writing‐original draft; writing‐review & editing.

## ETHICAL STATEMENT

The authors are accountable for all aspects of the work in ensuring that questions related to the accuracy or integrity of any part of the work are appropriately investigated and resolved. Written informed patient consent was obtained to publish this report.

## Supporting information


**Video S1** Surgical excision of the subpleural lesion with a nonintubated uniportal video‐assisted thoracoscopic surgery approach.

https://drive.google.com/drive/folders/14G2TfTybZNuP3CPtZ2E9efEVyzzb_0Wk?usp=sharing

## Data Availability

Data sharing is not applicable to this article as no new data were created or analyzed in this study.
